# Drug-prescribing patterns during pregnancy in the tertiary care hospitals of Pakistan: a cross sectional study

**DOI:** 10.1186/1471-2393-8-24

**Published:** 2008-07-15

**Authors:** Dileep K Rohra, Nirmal Das, Syed I Azam, Nazir A Solangi, Zahida Memon, Abdul M Shaikh, Nusrat H Khan

**Affiliations:** 1 Department of Biological & Biomedical Sciences, Aga Khan University, Stadium Road, Karachi, Pakistan; 2 Department of Pharmacology & Therapeutics, Bolan Medical College, Quetta, Pakistan; 3 Department of Community Health Sciences, Aga Khan University, Stadium Road, Karachi, Pakistan; 4 Department of Pharmacology & Therapeutics, Nawabshah Medical College, Nawabshah, Pakistan; 5 Department of Pharmacology & Therapeutics, Dow University of Health Sciences, Karachi, Pakistan; 6 Department of Pharmacology & Therapeutics, Chandka Medical College, Larkana, Pakistan; 7 Department of Obstetrics & Gynaecology, Unit III, Dow Medical College and Civil Hospital, Karachi, Pakistan

## Abstract

**Background:**

The rationale for use of drugs during pregnancy requires a careful assessment as in addition to the mother, the health and life of her unborn child is also at stake. Information on the use of drugs during pregnancy is not available in Pakistan. The aim of this study was to evaluate the patterns of drug prescriptions to pregnant women in tertiary care hospitals of Pakistan.

**Methods:**

This was a cross-sectional study conducted at five tertiary care hospitals of Pakistan. Copies of outpatient medicinal prescriptions given to pregnant patients attending the antenatal clinics were collected. The drugs were classified according to the pharmacological class and their teratogenic potential.

**Results:**

All the pregnant women attending the antenatal clinics received a prescription containing at least one drug. A total of 3769 distinct prescriptions given to different women were collected. Majority of the women who received the prescriptions belonged to third trimester (55.4%) followed by second (33.6%) and first trimester (11.0%). On an average, each prescription contained 1.66 ± 0.14 drugs. The obstetricians at Civil Hospital, Karachi and Chandka Medical College Hospital, Larkana showed a tendency of prescribing lesser number of drugs compared to those in other hospitals. Anti-anemic drugs including iron preparations and vitamin and mineral supplements (79.4%) were the most frequently prescribed drugs followed by analgesics (6.2%) and anti-bacterials (2.2%). 739 women (19.6%) received prescriptions containing drugs other than vitamin or mineral supplements. Only 1275 (21.6%) of all the prescribed drugs (n = 6100) were outside this vitamin/mineral supplement class. Out of these 1275 drugs, 29 (2.3%) drugs were prescribed which are considered to be teratogenic. Misoprostol was the most frequently prescribed (n = 6) among the teratogenic drugs followed by carbimazole (n = 5) and methotrexate (n = 5). Twenty nine pregnant women (0.8% of all the women studied) were prescribed these teratogenic drugs.

**Conclusion:**

Less than one percent of the pregnant women attending tertiary care hospitals in Pakistan are prescribed teratogenic drugs. The prescribing practices of Pakistani physicians are similar to those in western countries.

## Background

Careful consideration of the benefit to the mother and the risk to the fetus is required while prescribing drugs during pregnancy. Reducing medication errors and improving patient safety are the important areas of discussion [1]. The use of drugs during pregnancy calls for special attention because in this case in addition to the mother, the health and life of her unborn child is also at stake. The drugs given to pregnant mothers for therapeutic purposes may cause serious structural and functional adverse effects in the developing child [2]. Since it is very difficult to determine the effects on the fetus before marketing new drugs due to obvious ethical reasons, most drugs are not recommended to be used during pregnancy [3].

Since there are numerous gaps in knowledge about deleterious consequences for the fetus, prescription drug use by pregnant women should be viewed as a public health issue [4]. Pharmacoepidemiological studies can measure the extent of prescription and teratogenic drug use in pregnant women. The studies conducted in developed countries where drug-prescribing practices are considered to be superior, have identified need for interventional measures aimed at rational prescription during the prenatal period [5,6]. Similar type of pharmacoepidemiological studies auditing the use of drugs during pregnancy have not been conducted in Pakistan. Hence the aim of this study was to examine the patterns of drug prescription during pregnancy in the tertiary care hospitals of Pakistan. With this information, we intend to provide feedback and recommendations for the health care providers.

## Methods

This was a cross-sectional study conducted at five tertiary care hospitals of Pakistan i.e Aga Khan university Hospital (AKUH), Karachi, Civil Hospital (CH), Karachi; Nawabshah Medical College Hospital (NMCH), Nawabshah; Chandka Medical College Hospital (CMCH), Larkana and Bolan Medical College Hospital (BMCH), Quetta. The sampling units were carefully selected so as to get the data that can maximally represent the prescribing trends in Pakistan. Briefly, the sampling units were a mix of public sector (CH, NMCH, BMCH and CMCH) and private sector (AKUH). It was also a mix of metropolitan (AKUH and CH) and smaller town hospitals (CMCH, NMCH and BMCH). Ethical clearance was obtained from the Ethical Review Committee of the Aga Khan University, Karachi. In addition to that a written clearance was obtained form the Heads of the sampling units for obtaining the data from their respective Hospitals. Copies of outpatient drug prescriptions given to pregnant patients attending the antenatal clinics of CH, NMCH, CMCH, and BMCH were collected by the data collectors from January 1 to April 30, 2007. Before making copies of the prescription, verbal consent was obtained from the women whom prescriptions were issued. Since the AKUH maintains a record of all the patients attending the clinics, data was collected from the medical records of the pregnant women attending the antenatal clinics during the above-mentioned period. In order to prevent bias, the prescribers were kept unaware about the collection of prescriptions. The prescriptions given to women on repeat visits were not included, so effectively all the prescriptions analyzed in this study represent different women.

Data was recorded on a structured questionnaire that contained information on the demography of patients, brand names of the drugs prescribed and number of drugs written in each prescription.

All the completed questionnaires were pooled together. The generic names and then the pharmacological class of each drug prescribed to pregnant women were identified by a drug data base published every year in Pakistan [7].

The collected data was entered and analyzed using statistical software SPSS (version 15.0). One way ANOVA was used to compare the number of drugs prescribed in each sampling unit.

## Results

All the pregnant women attending the antenatal clinics of all five sampling units received a prescription containing at least one drug during their visits. A total of 3769 prescriptions issued to the same number of women were collected. The number of prescriptions collected from all five centers was more or less in the similar range. The maximum (879) and minimum (601) number of prescriptions were collected from CMCH and CH, respectively. The mean age of the women under study was 28.3 ± 2.4 years. The distribution of gestational age of the women in all sampling units whose prescriptions were collected is presented in Table 1. As evident from Table 1, the maximum number of prescriptions was collected from women in their third trimester of pregnancy (55.4%) followed by second (33.6%) and first trimester (11.0%).

**Table 1:** Trimester-wise break up of patients attending the antenatal clinics, whose prescriptions were collected.

**Trimester of Drug Prescription**	**Sampling Unit**					**Total**
	
	**AKUH**	**CH**	**CMCH**	**BMCH**	**NMCH**	
		
	**n (%)**	**n (%)**	**n (%)**	**n (%)**	**n (%)**	**n (%)**
First trimester	16 (2.3)	29 (4.8)	128 (14.6)	132 (17.4)	105 (13.3)	**410 (11.0)**
Second trimester	203 (29.0)	158 (26.4)	287 (32.8)	331 (43.6)	271 (34.3)	**1250 (33.6)**
Third trimester	482 (68.8)	412 (68.8)	460 (52.6)	296 (39.0)	415 (52.5)	**2065 (55.4)**
Did not specify*	10	2	4	20	8	**44**
**Total**	**711 (18.9)**	**601 (15.9)**	**879 (23.3)**	**779 (20.7)**	**799 (21.2)**	**3769 (100.0)**

*Mean ± SEM of number of drugs prescribed*	1.84 ± 0.039	1.35 ± 0.023	1.39 ± 0.020	1.78 ± 0.027	1.92 ± 0.040	1.66 ± 0.014**
*Range*	1 – 10	1 – 5	1 – 4	1 – 6	1 – 8	**1 – 10**

*These observations are not included in the percentage distribution of total number of patients by hospitals.

** p-value < 0.001 (using One Way ANOVA) i.e. a significant association was observed between number of drugs prescribed and hospitals. CH and CMCH are prescribing lesser number of drugs as compared to other hospitals.

While analyzing the number of drugs prescribed to pregnant women, it was found out that on the average, each prescription contained 1.66 ± 0.14 drugs. Interestingly, the obstetricians at CH and the CMCH had the tendency of prescribing lesser number of drugs compared to AKUH, BMCH and NMCH, as shown in Table 1.

The pharmacological classes of drugs prescribed to pregnant women were also analyzed. As shown in Table 2, anti-anemic drugs including iron preparations and vitamin and mineral supplements (79.4%) were the most frequently prescribed drugs followed by analgesics (6.2%).

**Table 2:** Distribution of Drug Classes prescribed to pregnant women.

**Pharmacological Class**	**Gestational age (Trimester)**			
		
	**First n**	**Second n**	**Third n**	**Total n**
	**(%)**	**(%)**	**(%)**	**(%)**
Anti-anemic/vitamin and mineral supplements	491 (75.5)	1577 (78.8)	2775 (80.5)	4843 (79.4)
Analgesics (including opioids)	50 (7.7)	132 (6.6)	200 (5.8)	382 (6.2)
Anti-platelets/anti-coagulants	11 (1.7)	21 (1.1)	23 (0.7)	55 (0.9)
Estrogens/anti-estrogen	1 (0.6)	4 (0.4)	10 (0.9)	15 (0.2)
Progesterones	5 (0.8)	30 (1.5)	30 (0.9)	65 (1.1)
Corticosteroids (oral, injectable, inhalational and topical)	5 (0.8)	40 (2.0)	71 (2.1)	116 (1.9)
Antacids	6 (0.9)	32 (1.6)	34 (1.0)	72 (1.2)
Anti-histamines	8 (1.2)	10 (0.5)	18 (0.5)	36 (0.6)
Anti-emetics	31 (4.8)	30 (1.5)	32 (0.9)	93 (1.5)
Anti-bacterial (oral and topical)	10 (1.5)	32 (1.6)	91 (2.6)	133 (2.2)
Anti-fungal (oral and topical)	0 (0.0)	2 (0.1)	21 (0.6)	23 (0.4)
Anti-helminthic	2 (0.3)	3 (0.1)	13 (0.4)	18 (0.3)
Anti-asthmatic (oral and inhalational)	5 (0.8)	13 (0.6)	17 (0.5)	35 (0.6)
Anti-cholinergics	12 (1.8)	49 (2.4)	73 (2.1)	134 (2.2)
CNS drugs including anti- epileptics	4 (0.6)	9 (0.4)	13 (0.4)	26 (0.4)
Miscellaneous	9 (1.4)	18 (0.9)	27 (0.8)	54 (0.9)
				
**Total**	**650 (100.0)**	**2002 (100.0**)	**3448 (100.0)**	**6100 (100.0)**

Further analysis was done to determine the extent of prescription of potentially teratogenic dugs. In this analysis, we did not include the vitamin and mineral supplements prescribed to pregnant women. Out of 3769 women included in this study, 739 (19.6%) received prescriptions containing drugs other than vitamin or mineral supplements. Only 1275 (21.6%) of all the prescribed drugs (n = 6100) were outside this vitamin/mineral supplement class. Out of these 1275 drugs, 29 (2.3%) drugs were prescribed which are considered to have teratogenic potential. Twenty nine pregnant women (0.8% of all the women studied) were prescribed these teratogenic drugs. Table 3 depicts the distribution of teratogenic drugs prescribed in the present study. Misoprostol was the most frequently prescribed (n = 6) among the teratogenic drugs followed by carbimazole (n = 5) and methotrexate (n = 5).

**Table 3:** Distribution of teratogenic drugs prescribed to pregnant women.

**Name of the drug**	**n**
Misoprostol	6
Carbimazole	5
Methotrexate	5
Warfarin	4
Carbamazepine	4
Phenytoin	3
Valproate sodium	2

**Total**	**29**

## Discussion

Although a number of similar studies have been conducted in the western countries, this is the first one conducted in Pakistan which has determined the prescribing attitudes of antenatal care providers in tertiary care hospitals. Furthermore, this study has determined the extent of prescription of drugs which are considered teratogenic for the fetus. To the best of our knowledge, there is only one other study in South Asia that has examined the prescribing behavior of physicians in pregnant women in India, focusing on the pharmacological class of the drugs and their safety profile [8]. The strength of the present study is that the determination of exposure of drugs was based on physical prescriptions rather than on recall, which may lead to bias or underascertainment [9].

Based on limited reported effects in humans and more extensive studies with animals, different classification systems have been made. Swedish system was the first to be implemented in 1978 [10]. Later on, US Food and Drug Administration (FDA) classified the drugs into categories based on the risk of induction of fetal toxicity. Many previous studies have used these risk classification systems as the tools to evaluate the prescribing behaviors of the physicians to pregnant women [8,11-13]. However, there are some studies which have questioned the validity of these classification systems [2,14]. Therefore, we focused our analysis on the drugs which are documented as teratogenic in humans. Published studies that evaluate drug use during pregnancy have used varying methodologies including the collection of prescriptions [15], interview of mothers [16], and use of pharmacy records [17]. Furthermore, the analysis of drug use in those studies has been done based on various risk classification systems, which makes the comparison of our results with those studies difficult. However, the proportion of pregnant women who were prescribed potentially teratogenic drugs (0.8%) is comparable to the recently published results of Andrade et al (2006), who have reported that 1.1% pregnant population in United States received a teratogenic drug [18]. We understand that ours is a snap shot study which has analyzed the prescriptions issued to women during pregnancy. This is exactly in accordance with the objective of the study which was to evaluate the prescribing trends of Pakistani physicians. However, the actual exposure to potentially teratogenic drugs could well be different. This is especially in the context of Pakistan where most of the pregnancies are unplanned. The pregnant women might be taking drugs before they know that they have become pregnant.

There are certain situations where the doctors are left with no option but to prescribe teratogenic drugs when a suitable replacement is not available and/or when the benefits of the drug to the mother outweigh the possible risk to the fetus. The use of anti-epileptic drugs is one good example in this context [19]. However, anecdotal reports suggest an alarming trend in Pakistan that many doctors are prescribing the drugs without knowing their significant toxic effects, drug interaction and contraindications. Based on those reports, we expected very high rate of prescription of potentially teratogenic drugs. Contrary to our expectation, this study revealed a rather careful prescribing behaviour of the physicians to pregnant women. The percentage of women who were prescribed teratogenic drugs was comparable to that reported in USA [18].

Another observation from the current study was the relatively smaller number of women in their first trimester, who were recruited. We tried to recruit all pregnant women attending the clinics, but the proportion of them in their first trimester was less in all the five sampling units. The less number of women from first trimester in our study is perhaps because of the fact that pregnant women in Pakistan start seeking antenatal care in advanced pregnancy.

It is emphasized that the results of the present study can reasonably be considered as the prescribing trends prevalent in the tertiary care hospitals of the whole of Pakistan because of the representativeness of the sampling units. The five sampling units of the study covered four cities and towns of two (out of four) provinces with a wide ethnic, socioeconomic and geographical variations. Nonetheless, the results from all the hospitals were almost similar in terms of prescribing patterns of the physicians.

## Conclusion

Less than one percent of the pregnant women attending tertiary care hospitals in Pakistan are prescribed teratogenic drugs. The prescribing practices of Pakistani physicians are similar to those in western countries.

## Competing interests

The authors declare that they have no competing interests.

## Authors' contributions

DKR: Principal Investigator. Conceptualized the study, received the funding, supervised the project and wrote the manuscript. ND: Co-Principal Investigator. Provided intellectual input, supervised the project at the Bolan Medical College Hospital, Quetta and reviewed the manuscript. SIA: Co-Principal Investigator. Provided intellectual input for the methodology, did the statistical analysis and reviewed the manuscript. NAS: Co-Principal Investigator. Provided intellectual input, supervised the project at the Nawabshah Medical College Hospital, Nawabshah and reviewed the manuscript. ZM: Co-Principal Investigator. Provided intellectual input, supervised the project at the Civil Hospital, Karachi and reviewed the manuscript. AMS: Co-Principal Investigator. Provided intellectual input, supervised the project at the Chandka Medical College Hospital, Larkana and reviewed the manuscript. NHK: Co-Principal Investigator. Provided intellectual input, supervised the project at the Civil Hospital, Karachi and reviewed the manuscript.

## Pre-publication history

The pre-publication history for this paper can be accessed here:



## References

[B1] Benjamin DM (2003). Reducing medication errors and increasing patient safety: case studies in clinical pharmacology. J Clin Pharmacol.

[B2] Kacew S (1994). Fetal consequences and risks attributed to the use of prescribed and over-the-counter (OTC) preparations during pregnancy. Int J Clin Pharmacol Ther.

[B3] Koren G, Pastuszak A, Ito S (1998). Drugs in pregnancy. N Eng J Med.

[B4] De Jong-van den Berg LT, Berg PB Van den, Haaijer-Ruskamp FM, Dukes MN, Wesseling H (1991). Investigating drug use in pregnancy. Methodological problems and perspectives. Pharm Weekbl Sci.

[B5] Beyens MN, Guy C, Ratrema M, Ollagnier M (2003). Prescription of drugs to pregnant women in France: the HIMAGE study. Therapie.

[B6] (1992). Medication during pregnancy: an intercontinental cooperative study. Collaborative Group on Drug Use in Pregnancy (C.G.D.U.P.). Int J Gynaecol Obstet.

[B7] Pakistan Drug Index Ready reference handbook of all medicines available in Pakistan.

[B8] Sharma R, Kapoor B, Verma U (2006). Drug utilization pattern during pregnancy in North India. Indian J Med Sci.

[B9] Rockenbauer M, Olsen J, Czeizel AE, Pedersen L, Sørensen HT, EuroMAP Group (2001). Recall bias in a case-control surveillance system on the use of medicine during pregnancy. Epidemiology.

[B10] Sannerstedt R, Lundborg P, Danielsson BR, Kihlström I, Alván G, Prame B, Ridley E (1996). Drugs during pregnancy: an issue of risk classification and information to prescribers. Drug Saf.

[B11] Lacroix I, Damase-Michel C, Lapeyre-Mestre M, Montastruc JL (2000). Prescription of drugs during pregnancy in France. Lancet.

[B12] Cooper WO, Hickson GB, Ray WA (2004). Prescriptions for contraindicated category X drugs in pregnancy among women enrolled in TennCare. Paed Perinatal Eidemiol.

[B13] Riley EH, Fuentes-Afflick E, Jackson RA, Escobar GJ, Brawarsky P, Schreiber M, Haas JS (2005). Correlates of prescription drug use during pregnancy. J Women's Health.

[B14] Addis A, Sharabi S, Bonati M (2000). Risk classification systems for drug use during pregnancy: are they a reliable source of information?. Drug Saf.

[B15] Das B, Sarkar C, Datta A, Bohra S (2003). A study of drug use during pregnancy in a teaching hospital in western Nepal. Pharmacoepidemiol Drug Saf.

[B16] Glover DD, Amonkar M, Rybeck BF, Tracy TS (2003). Prescription, over-the-counter, and herbal medicine use in a rural, obstetric population. Am J Obstet Gynecol.

[B17] De Jong-van den Berg LT, Berg PB Van den, Haaijer-Ruskamp FM, Dukes MN, Wesseling H (1991). Investigating drug use in pregnancy. Methodological problems and perspectives. Pharm Weekbl Sci.

[B18] Andrade SE, Raebel MA, Morse AN, Davis RL, Chan KA, Finkelstein JA, Fortman KK, McPhillips H, Roblin D, Smith DH, Yood MU, Platt R, H Gurwitz J (2006). Use of prescription medications with a potential for fetal harm among pregnant women. Pharmacoepidemiol Drug Saf.

[B19] Eadie MJ (2008). Antiepileptic drugs as human teratogens. Expert Opin Drug Saf.

